# m6A RNA methylation regulators could contribute to the occurrence of chronic obstructive pulmonary disease

**DOI:** 10.1111/jcmm.15848

**Published:** 2020-09-22

**Authors:** Xinwei Huang, Dongjin Lv, Xiao Yang, Min Li, Hong Zhang

**Affiliations:** ^1^ Translational Research Institute of Brain and Brain‐Like Intelligence Shanghai Fourth People’s Hospital Affiliated to Tongji University School of Medicine Shanghai China; ^2^ Department of Rehabilitation Medicine Shanghai Fourth People's Hospital Affiliated to Tongji University School of Medicine Shanghai China; ^3^ Department of Medical Oncology The Third Affiliated Hospital of Kunming Medical University (Tumor Hospital of Yunnan Province) Kunming China

**Keywords:** bioinformatics, chronic obstructive pulmonary disease, IGF2BP3, m6A RNA methylation regulators, METTL3

## Abstract

N6‐methyladenosine (m6A) RNA methylation, the most prevalent internal chemical modification of mRNA, has been reported to participate in the progression of various tumours via the dynamic regulation of m6A RNA methylation regulators. However, the role of m6A RNA methylation regulators in chronic obstructive pulmonary disease (COPD) has never been reported. This study aimed to determine the expression and potential functions of m6A RNA methylation regulators in COPD. Four gene expression data sets were acquired from Gene Expression Omnibus. Gene ontology function, Kyoto Encyclopedia of Genes and Genomes pathway enrichment analyses, weighted correlation network analysis and protein‐protein interaction network analysis were performed. The correlation analyses of m6A RNA methylation regulators and key COPD genes were also performed. We found that the mRNA expressions of IGF2BP3, FTO, METTL3 and YTHDC2, which have the significant associations with some key genes enriched in the signalling pathway and biological processes that promote the development progression of COPD, are highly correlated with the occurrence of COPD. In conclusion, six central m6A RNA methylation regulators could contribute to the occurrence of COPD. This study provides important evidence for further examination of the role of m6A RNA methylation in COPD.

## 1 INTRODUCTION

Chronic obstructive pulmonary disease (COPD) is characterized by a progressive condition of small airway obstruction, chronic bronchitis and emphysema, representing a leading cause of death worldwide.^1^ Although oxidative stress, immunity and inflammation, apoptosis, metaplastic epithelial lesions, mucus hypersecretion and fibrosis in both airways and lungs are hallmarks of this disease,^1^, ^2^ the exact pathogenesis of COPD remains unclear.

To date, more than 100 kinds of RNA modifications have been identified in living organisms.^3^ Certain types of RNA modifications in eukaryotic mRNA, such as 5‐methylcytosine (m5C), N1‐methyladenosine (m1A) and N6‐methyladenosine (m6A), have widely discovered,^4^ among which m6A RNA methylation was first discovered in the 1970s.^5^ The m6A RNA methylation, the most common form of dynamic and reversible mRNA modification, is regulated by m6A RNA methylation regulators, containing methyltransferases (‘writers’) that install m6A mark, demethylases (‘erasers’) that reverse m6A mark, and m6A binding proteins (‘readers’) that decode m6A modification to mediate the fate of modified transcripts.^6^, ^7^, ^8^ Currently, at least ten proteins have been found in the m6A ‘writers’ complex, containing WT1‐associated protein (WTAP), methyltransferase like 3/4/14 (METTL3/4/14), Cbl Proto‐Oncogene Like 1 (CBLL1), RNA‐binding motif protein15/15B (RBM15/15B), Vir like m6A methyltransferase associated VIRMA (VIRMA/KIAA1429), Zinc Finger Protein 217 (ZNF217) and zinc finger CCCH domain‐containing protein 13 (ZC3H13).^3^, ^4^, ^6^, ^8^, ^9^ ‘Erasers’ consist of fat mass alkB homolog 5 (ALKBH5) and obesity‐associated protein (FTO).^9^ Twelve proteins have been identified in the m6A ‘readers’, including YT521‐B homology (YTH) domain‐containing proteins (YTHDC1/2), eukaryotic translation initiation factor 3 subunit A/B (EIF3A/B), YTH N6‐methyladenosine RNA‐binding proteins (YTHDF1/2/3), insulin like growth factor 2 mRNA binding protein families (IGF2BP1/2/3) and heterogeneous nuclear ribonucleoprotein (HNRNP) protein families (HNRNPA2B1 and HNRNPC).^3^, ^4^, ^6^, ^8^, ^9^

The m6A RNA methylation modulated by these regulators has been found to influence mRNA at different levels, containing structure, maturation, splicing, nuclear export, translation, localization, stability and decay,^3^, ^4^, ^6^, ^7^, ^8^, ^9^ playing important roles in diverse physiological processes, including development, fertility, stemness, meiosis, carcinogenesis, circadian cycle, cell fate decision, cell differentiation, cell cycle regulation and circadian rhythm maintenance and human diseases.^6^, ^8^, ^10^ Particularly, an increasing number of in vivo and in vitro experiments and bioinformatic researches showed that m6A RNA methylation regulators contribute to the occurrence, development and clinical prognosis of multiple various cancers,^4^, ^6^, ^7^, ^8^, ^9^, ^11^, ^12^, ^13^, ^14^ while the role of m6A RNA methylation regulators in COPD and their association with COPD genes are never reported.

In the current study, we first explored and validated key m6A RNA methylation regulators that were significantly correlated with the occurrence of COPD and determined key COPD genes. In addition, we further analysed the relevance of m6A RNA methylation regulators with these key genes.

## 2 METHODS

### 2.1 Data acquisition and preprocessing

Four gene array expression Series Matrix Files of small airway epithelium samples, containing GSE5058, GSE8545, GSE11906 and GSE20257, were obtained from the Gene Expression Omnibus data sets (https://www.ncbi.nlm.nih.gov/geo/). GSE5058 includes molecular profiles of 15 samples from COPD cases and 24 samples from healthy controls.^15^ GSE8545 contains expression profiles of 54 samples, including 18 COPD cases and 36 healthy controls.^16^ GSE11906 includes 33 samples from COPD cases and 98 from healthy controls.^17^ GSE20257 contains 23 samples from COPD cases and 112 from healthy controls.^18^ Their experiments were conducted on the Affymetrix Human Genome U133 Plus 2.0 Array (GPL570 platform, Affymetrix, Inc). Gene symbols of data sets were obtained using the R software and annotation packages. Subsequently, the log2 converted and standardized mRNA expression data were obtained by R 3.61 and limma package 3.40.6. Given the limited sample size of small airway epithelium in COPD, we merged and batch‐normalized GSE8545 and GSE20257 into the train group using R packages (sva and limma3.40.6). Validation group 1 was obtained from the merged and batch‐normalized GSE5058 and GSE11906, and validation group 2 was gained from the merged and batch‐normalized four data sets. A flow chart of this study was showed in Figure S1.

### 2.2 Selection of m6A RNA methylation regulators

Twenty‐four widely recognized m6A RNA methylation regulators were collated from published literature.^3^, ^4^, ^6^, ^8^, ^9^ We then obtained their mRNA expression data from the train group, validation group 1 and validation group 2, respectively. Differential expression analysis for 24 m6A RNA methylation regulators between COPD cases and health controls was performed using wilcox.test. R package corrplot was used to identify the correlation between m6A RNA methylation regulators. The *P* value < .05 was considered as statistical significant.

### 2.3 Identification of co‐expression modules and key COPD genes

Firstly, the differentially expressed genes (DEGs) between COPD cases and health controls for train group, validation group 1 and validation group 2, were separately identified by the limma package 3.40.6. The *P* value < .05 after being corrected by false discovery rate (FDR) and |log2 fold change (FC)| > 1 were applied as the cut‐off for DEGs screening. The overlapped DEGs were obtained from the above three groups.

Subsequently, we used differentially expressed genes (FDR < 0.05) obtained from train group to identify the hub COPD genes by weighted correlation network analysis (WGCNA) package on mRNA expression data of validation group 1. The detailed procedure and introduction of WGCNA were shown in WGCNA R package.^19^ In brief, a weighted adjacency matrix was first constructed based on the selected soft threshold power. Subsequently, the connectivity measure per gene was calculated according to the connection strengths with other genes. Then, the module structure preservation was identified by module preservation R function. Finally, the gene expression profiles of each module were summarized by the module eigengene, and each module eigengene was regressed on COPD trait using the linear model in the limma R package.^20^

Finally, key COPD genes were acquired by taking the intersection of the overlapped DEGs and hub COPD genes.

### 2.4 Bioinformatic analysis

The interactions among 24 m6A RNA methylation regulators and key COPD genes were analysed using the STRING database (http://www.string‐db.org/) and were visualized by Cytoscape 3.7.1.^21^ Kyoto Encyclopedia of Genes and Genomes (KEGG) pathway enrichment and Gene Ontology (GO) functional analyses were performed to analyse 24 m6A RNA methylation regulators and key COPD genes using R packages (clusterProfiler, org.Hs.eg.db, enrichplot, colorspace, stringi and ggplot2). The *P* value < .05 was applied as the cut‐off.

### 2.5 The Correlation of m6A RNA methylation regulators with key COPD genes

R package ggpubr was used to determine the correlation between m6A RNA methylation regulators and key COPD genes in the COPD data of validation group 2. The *P* value < .05 was considered as statistical significant.

## 3 RESULTS

### 3.1 Expression of m6A RNA methylation regulators in COPD

**FIGURE 1:**
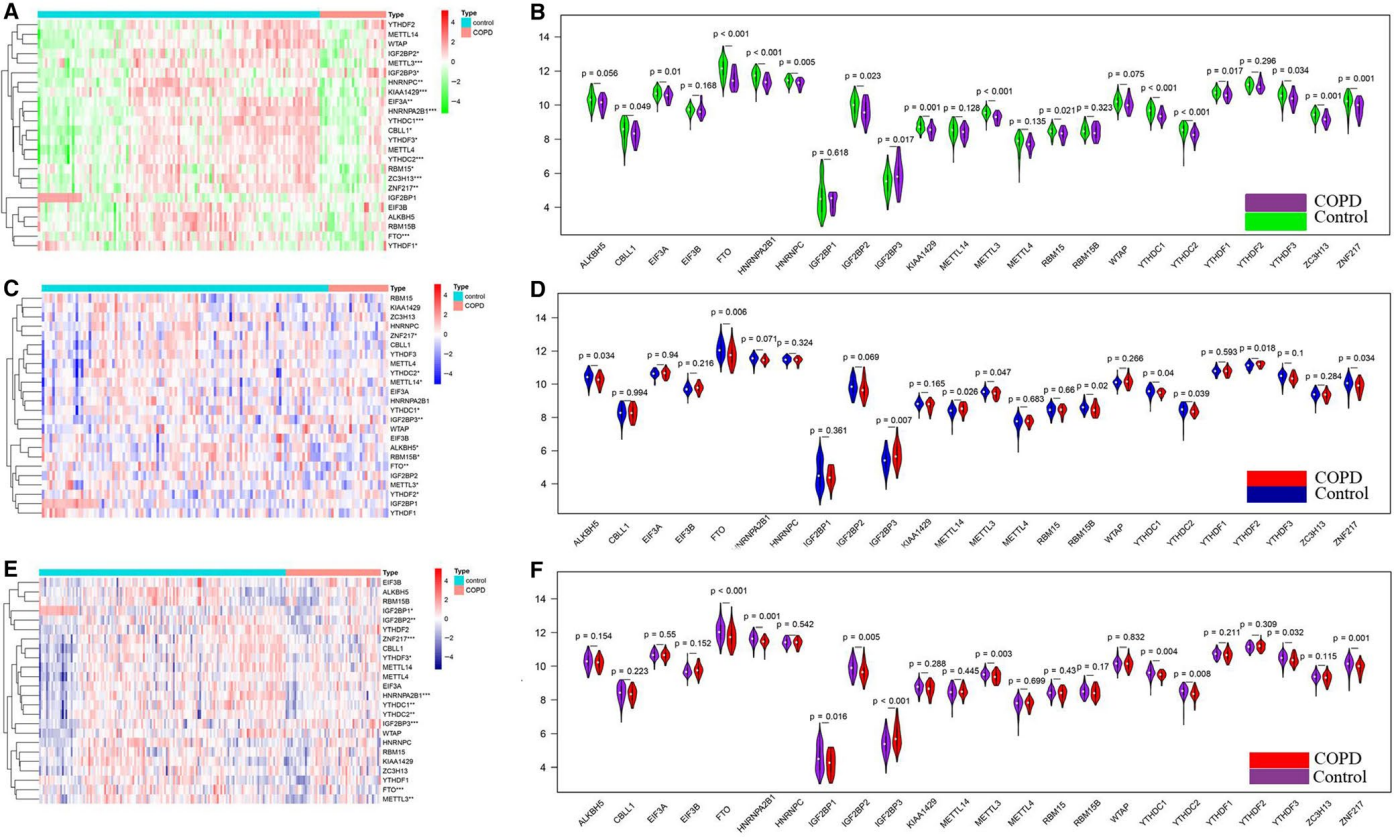
Expression of m6A RNA methylation regulators between healthy controls and COPD cases. A and B, Heat map and violin diagram of expression levels of 24 m6A RNA methylation regulators (healthy control sample vs COPD sample) from train group. C and D, Heat map and violin diagram of expression levels of 24 m6A RNA methylation regulators from validation group 1. E, F, Heat map and violin diagram of expression levels of 24 m6A RNA methylation regulators from validation group 2

Firstly, the differential expression analysis for 24 m6A RNA methylation regulators between the COPD samples and control samples was performed in train group, we found that 16 m6A RNA methylation regulators were significantly associated with the occurrence of COPD (Figure 1A), with 15 down‐regulated and 1 up‐regulated genes in COPD samples comparing to control samples (Figure 1B). Subsequently, the significant association between COPD occurrence and the expression of IGF2BP3, FTO, ZNF217, METTL3, YTHDC1 and YTHDC2 was confirmed in both validation group 1 and validation group 2 (Figure 1C‐F). Compared with the controls samples, the expression of FTO, ZNF217, METTL3, YTHDC1 and YTHDC2 was down‐regulated, while the expression of IGF2BP3 was up‐regulated in COPD.

**FIGURE 2:**
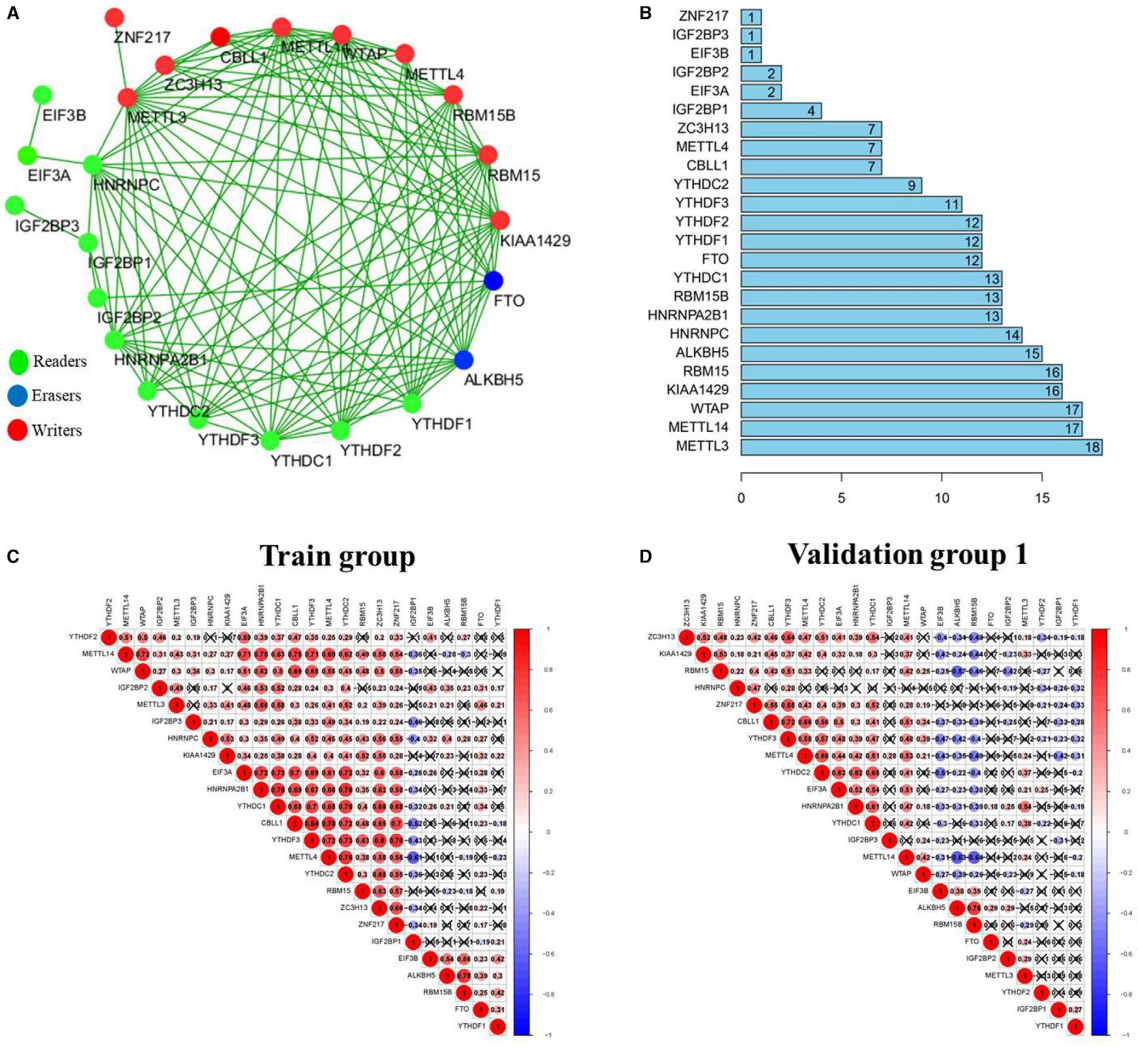
Correlation among the expression of m6A methylation regulators. A, The protein‐protein interactions among 24 m6A methylation regulators. B, The rank of connection degree (number) for each gene. C and D, Correlation among the expression of 24 m6A methylation regulators in train group and validation group 1

To determine the interactions among 24 m6A RNA methylation regulators, we performed an analysis of protein‐protein interaction (PPI) networks. The results showed that these writers, erasers and readers frequently interacted with each other (Figure 2A) and presented an especially high degree of connections among the writers (METTL3, METTL14, WTAP, KIAA1429 and RBM15) (Figure 2B), indicating that writers, erasers and readers do not function in isolation, but exists collaboration. Therefore, we further investigated the expression correlation among these writers, erasers and readers. We observed that a highly consistent correlation existed among m6A RNA methylation regulators in both train group and validation group 1 (Figure 2C, D). For instance, the writers (METTL4, METTL14, RBM15B, ZC3H13, ZNF217 and CBLL1), eraser ALKBH5 and readers (IGF2BP3, YTHDF1/3, YTHDC1/2 and EIF3A/B) were significantly associated with other m6A RNA methylation regulators (0.19 < |R| < 0.80 and *P* value < .05). Taken together, these results indicated that cross‐talk among the writers, erasers and readers of m6A RNA methylation regulators may have important role in the occurrence of COPD.

### 3.2 Identification of DEGs in COPD

As shown in Volcano plots (Figure S2A‐C), under the thresholds of |log2FC| > 1 and FDR < 0.05, a total of 93 DEGs including 43 down‐regulated and 50 up‐regulated genes were obtained from train group. Seven down‐regulated and 39 up‐regulated DEGs were gained from validation group 1. Validation group 2 contained 39 up‐regulated and 10 down‐regulated DEGs. Among these DEGs, 29 up‐regulated genes and 3 down‐regulated genes were overlapped among three groups (Figure S2D).

### 3.3 Identification of co‐expression modules and key COPD genes

In order to determine the modules and genes correlated with the occurrence of COPD, we performed WGCNA for 4410 genes obtained from train group according to the thresholds of FDR < 0.05 on validation group 1. The lowest soft threshold power 6, for which the scale‐free topology fit index reaches 0.90, was used to conduct a hierarchical clustering tree. Subsequently, nine modules were generated, including black, blue, brown, green, greenyellow, grey, magenta, pink and yellow modules. The grey module, including the non‐co‐expressed genes, was not further analysed (Figure S3A,B). To identify modules correlated with COPD status, we analysed the relationship between each module and COPD. Form Figure S3C, we could conclude that the modules of black, green, greenyellow, magenta and yellow revealed the strongest significant correlations with COPD, indicating that genes in these modules are predominantly correlated with this disease. Based on the criteria that cor.COPD > 0.2 and cor.module membership > 0.6, a total of 336 genes with the high connectivity in black, green, greenyellow, magenta and yellow modules were screened as hub genes (Figure S3D‐H).

Finally, 27 COPD key genes were acquired by taking the intersection of 32 overlapped DEGs and 336 hub COPD genes (Table 1). Among the above 27 key COPD genes, except that LTF and SEMA5B expressions were notably reduced in COPD and negatively associated with COPD, the expression of other genes was notably up‐regulated and positively correlated with this disease.

**Table 1:** Twenty‐seven key differentially expressed gene positively or negatively associated with COPD and each MM

Key genes	Module colour	Cor. COPD	*P*.GS.COPD	Cor.MM	*P*.MM	Up or down in three groups
BCL2A1	Black	0.33635796	7.54E‐05	0.903687415	4.27E‐50	Up
CLEC5A	Black	0.37472462	8.84E‐06	0.738236674	3.73E‐24	Up
CCL2	Black	0.39099825	3.27E‐06	0.73301268	1.13E‐23	Up
MMP12	Black	0.41024854	9.38E‐07	0.713224459	5.89E‐22	Up
PROK2	Black	0.28582547	0.000852	0.677410186	3.45E‐19	Up
PLA2G7	Black	0.42014752	4.79E‐07	0.662673241	3.69E‐18	Up
AKR1B10	Green	0.37548358	8.45E‐06	−0.926579463	1.74E‐57	Up
CABYR	Green	0.41976641	4.92E‐07	−0.919968248	3.96E‐55	Up
SLC7A11	Green	0.43590719	1.57E‐07	−0.91364644	4.67E‐53	Up
CYP1B1	Green	0.44622784	7.32E‐08	−0.903934738	3.64E‐50	Up
MUCL1	Green	0.33876692	6.65E‐05	−0.902168494	1.13E‐49	Up
LOC284825	Green	0.40937316	9.95E‐07	−0.895803983	5.68E‐48	Up
GPX2	Green	0.34350277	5.17E‐05	−0.886770489	9.72E‐46	Up
UCHL1	Green	0.34037623	6.10E‐05	−0.8700699	4.53E‐42	Up
ST3GAL4‐AS1	Green	0.38337326	5.24E‐06	−0.84835127	5.39E‐38	Up
ALDH3A1	Green	0.31097422	0.000269	−0.831591653	2.91E‐35	Up
LOC344887	Green	0.34864183	3.91E‐05	−0.824570447	3.31E‐34	Up
AHRR	Green	0.48551789	3.16E‐09	−0.780096563	1.86E‐28	Up
GAD1	Green	0.40581083	1.26E‐06	−0.773455497	1.03E‐27	Up
EGF	Green	0.38090973	6.09E‐06	−0.729019627	2.57E‐23	Up
CYP1A1	Green	0.44206835	9.98E‐08	−0.728558756	2.83E‐23	Up
SPP1	Green	0.36910632	1.23E‐05	−0.682284662	1.53E‐19	Down
LTF	Green	−0.3828154	5.43E‐06	0.680352729	2.12E‐19	Up
STATH	Greenyellow	0.35683960	2.49E‐05	0.620691106	1.60E‐15	Up
TCN1	Greenyellow	0.44571305	7.61E‐08	0.832577273	2.05E‐35	Up
CEACAM5	Greenyellow	0.33658058	7.46E‐05	0.893164229	2.68E‐47	Up
SEMA5B	Yellow	−0.5144968	2.37E‐10	0.737015473	4.84E‐24	Down

### 3.4 KEGG and GO enrichment analyses

The results of GO analysis for 24 m6A RNA methylation regulators demonstrated that METTL3 was involved in four biological processes including gene expression, regulation of cytokine biosynthetic process, mRNA transport, and negative regulation of translation, and seven molecular functions. IGF2BP3 was involved in seven biological processes containing gene expression, mRNA methylation, mRNA processing, mRNA splicing, positive regulation of cap‐independent translational initiation, mRNA destabilization and primary miRNA processing, and four molecular functions. FTO was related to oxidative single‐stranded RNA demethylation and oxidative RNA demethylase activity. ZNF217 was associated with nucleic acid and protein binding. In addition, YTHDC1 and YTHDC2 were involved in N6‐methyladenosine‐containing RNA binding, poly (A) RNA binding, and nucleic acid and protein binding (Table S1).

GO and KEGG pathway enrichment analyses were performed for the above 27 key COPD genes to explore potential biological processes correlated with COPD. GO analysis showed that these genes were involved in twenty two biological processes, including secondary metabolic process (CYP1B1, CYP1A1, SLC7A11 and AKR1B10), xenobiotic metabolic process (AHRR, CYP1B1, CYP1A1 and ALDH3A1), retinol metabolic process (CYP1B1, CYP1A1 and AKR1B10), cellular response to xenobiotic stimulus (AHRR, CYP1B1, CYP1A1 and ALDH3A1), cellular lipid catabolic process (CYP1B1, CYP1A1 and AKR1B10), response to toxic substance (CYP1B1, CYP1A1, SLC7A11, AKR1B10 and GPX2) and organic hydroxy compound metabolic process (CYP1B1, CYP1A1, SLC7A11 and AKR1B10) etc(Figure S4A), and fourteen molecular functions (Figure S4B), indicating that these key genes might play critical roles in the occurrence of COPD via influencing these biological processes and molecular functions. KEGG analysis suggested that metabolism of xenobiotics by cytochrome P450 (CYP1B1, CYP1A1 and ALDH3A1), an important signalling pathway that influences the xenobiotic metabolizing capability of the lung and the risk of developing of lung diseases,^22^ was associated with COPD.

### 3.5 The correlation between m6A RNA methylation regulators and key COPD genes

**FIGURE 3:**
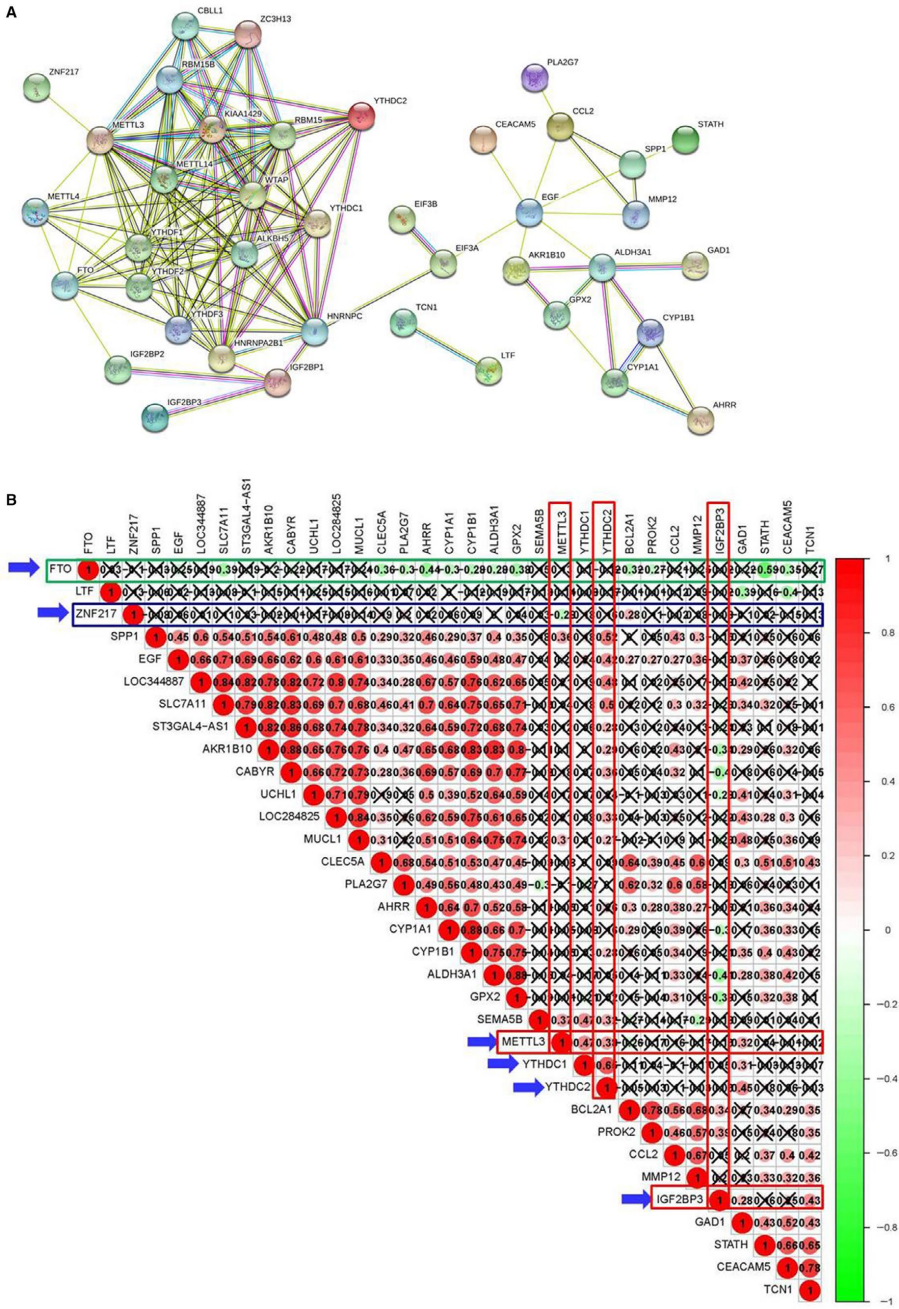
The correlation of IGF2BP3, FTO, ZNF217, METTL3, YTHDC1 and YTHDC2 with key COPD genes. A, The protein‐protein interactions among 24 m6A methylation regulators and 27 key COPD genes. B, Spearman correlation analyses between IGF2BP3, FTO, ZNF217, METTL3, YTHDC1 and YTHDC2 and key COPD genes

To further explore the roles of the six m6A RNA methylation regulators (IGF2BP3, FTO, ZNF217, METTL3, YTHDC1 and YTHDC2) and 27 key genes in COPD, we analysed the relevance between six m6A RNA methylation regulators and these key genes. PPI analysis indicated that these m6A RNA methylation regulators could indirectly interact with these key genes (Figure 3A). As shown in Figure 3B, the correlation analysis of expression in COPD data set of validation group 2 showed a positive association between most of 27 key COPD genes. FTO exhibited a negative correlation with the expression of SLC7A11, CLEC5A, PLA2G7, AHRR, CYP1B1, CYP1A1, ALDH3A1, GPX2, BCL2A1, PROK2, CEACAM5 and STATH (−0.59 < Cor < −0.28, *P* < .05). METTL3 showed a positive relationship with GAD1, SEMA5B, SPP1 and MUCL1 expressions (0.31 < Cor < 0.37, *P* < .05). IGF2BP3 expression was positively associated with GAD1, TCN1, BCL2A1, PROK2 expressions (0.34 < Cor < 0.43, *P* < .05) and negatively associated with GPX2, ALDH3A1, CYP1A1, UCHL1, CABYR and AKR1B10 expressions (−0.41 < Cor < −0.28, *P* < .05). Furthermore, YTHDC2 had a positive correlation with the expression of GAD1, SEMA5B, CYP1B1, MUCL1, LOC284825, CABYR, AKR1B10, ST3GAL4‐AS1, LOC344887, SLC7A11, EGF and SPP1 (0.27 < Cor < 0.52, *P* < .05). These results indicated that IGF2BP3, FTO, METTL3 and YTHDC2 could act as m6A RNA methylation regulators to influence these key expressions.

## 4 DISCUSSION

Abnormal m6A modification may cause the dysregulation of important genes that regulate crucial cellular processes and disrupt homeostasis, consequently leading to diseases.^23^ Numerous studies have demonstrated that m6A RNA methylation regulators contribute to the occurrence, development and clinical prognosis of multiple various cancers,^4^, ^6^, ^7^, ^8^, ^9^, ^11^, ^12^, ^13^, ^14^ due to its capacity of modulating RNA splicing, translocation, translation and stability. However, the understanding of m6A modification remains blank in COPD. This study was aimed to first explore the integrative roles of 24 common m6A RNA methylation regulators in COPD.

METTL3, a major catalytic enzyme of the m6A methyltransferase complex, plays a dual role (tumour suppressor or oncogene) in multiple human cancers, and regulates tumour cell proliferation, tumour formation migration, invasion and drug resistance.^24^ METTL3 was highly conserved in eukaryotes, and its knockdown in different cells significantly decreased m6A in mRNAs.^25^, ^26^, ^27^ ZNF217, an m6A methyltransferase inhibitor, interacts with some epigenetic regulators, activates the transcription of key pluripotency genes, regulates m6A deposition on their transcripts via sequestering METTL3 ^28^ and is involved in the proliferation, survival and invasiveness of cancer cells.^29^ FTO, serving as an m6A demethylase, primarily controls the m6A levels of downstream targets by their 3' untranslated regions and plays an important role in obesity‐related diseases and the occurrence, development and prognosis of multiple types of tumours.^30^ Acting as an m6A reader, YTHDC1 is implicated in the modulation of splicing by maintaining a dynamic interaction network of different family of proteins.^31^ YTHDC2 can promote a ‘fast‐track’ expression program for specific mRNAs by modulating the stability of m6A‐containing mRNAs and by facilitating their efficient translation.^32^ IGF2BP3, one of the bona fide oncofetal proteins, serves as a post‐transcriptional fine‐tuner in various human cancers, and regulates the expression of genes implicated in the modulation of tumour cell proliferation, metastasis, survival and chemo‐resistance.^33^ In current study, we first identified that IGF2BP3, FTO, ZNF217, METTL3, YTHDC1 and YTHDC2 were associated with the occurrence of COPD in three groups of data sets. PPI network and expression correlation analyses showed that cross‐talk among the writers, erasers and readers of m6A RNA methylation regulators had the critical role in the occurrence of COPD. Furthermore, we revealed that METTL3 might influence gene expression, regulation of cytokine biosynthetic process, mRNA transport and negative regulation of translation. IGF2BP3 could affect gene expression, mRNA methylation, mRNA processing, mRNA splicing, positive regulation of cap‐independent translational initiation, mRNA destabilization and primary miRNA processing. FTO was related to oxidative single‐stranded RNA demethylation and oxidative RNA demethylase activity. ZNF217 was associated with nucleic acid and protein binding. In addition, YTHDC1 and YTHDC2 were involved in N6‐methyladenosine‐containing RNA binding, poly (A) RNA binding, and nucleic acid and protein binding.

This study identified 27 key genes associated with the occurrence of COPD by WGCNA and differential expression analysis in three groups of data sets. These findings are consistent with the previous results that MUCL1, UCHL1, CABYR, CYP1B1, AHRR, AKR1B10, SLC7A11, ST3GAL4‐AS1, GPX2, LOC344887, EGF, CLEC5A, CCL2, MMP12, PLA2G7, GAD1, CYP1A1 and SPP1 were up‐regulated in a variety of samples from COPD or COPD‐related mice model, including peripheral blood mononuclear cells, large and small airway epithelium, quadriceps, blood, and the lung of mouse and human, as well as involved in the occurrence of COPD (Table S2).^15^, ^34^, ^35^, ^36^, ^37^, ^38^, ^39^, ^40^, ^41^, ^42^, ^43^, ^44^, ^45^, ^46^, ^47^, ^48^ GO and KEGG pathway enrichment analyses showed that CYP1B1, CYP1A1, SLC7A11, AKR1B10, AHRR, ALDH3A1 and GPX2 were involved in multiple biological processes related to metabolism of exogenous and endogenous stimulus, and metabolism of xenobiotics by cytochrome P450 signalling pathway, suggesting that the dysregulated expression of these genes might affect the development processes of COPD by influencing the above biological processes and signalling pathway. Notably, LOC284825, TCN1, STATH, CEACAM5, LTF and SEMA5B were first found to be associated with COPD in the current study.

In this study, the PPI network analyses between m6A RNA methylation regulators and key COPD genes showed that IGF2BP3, FTO, ZNF217, METTL3, YTHDC1 and YTHDC2 could indirectly interact with some key genes, such as CYP1B1, CYP1A1, AHRR, AKR1B10, GPX2, EGF,GAD1, CCL2, MMP12 and ALDH3A1. Moreover, the expression correlation analysis indicated a negative association of FTO expression with SLC7A11, CLEC5A, PLA2G7, AHRR, CYP1B1, CYP1A1, ALDH3A1, GPX2, BCL2A1, PROK2, CEACAM5 and STATH expressions. METTL3 showed a positive relationship with GAD1, SEMA5B, SPP1, and MUCL1 expressions. IGF2BP3 had a positive association with GAD1, TCN1, BCL2A1, PROK2 expressions and a negative association with GPX2, ALDH3A1, CYP1A1, UCHL1, CABYR and AKR1B10 expressions. Moreover, YTHDC2 had a positive correlation with the expression of GAD1, SEMA5B, CYP1B1, MUCL1, LOC284825, CABYR, AKR1B10, ST3GAL4‐AS1, LOC344887, SLC7A11, EGF and SPP1. These results indicated that IGF2BP3, FTO, METTL3 and YTHDC2 could act as m6A RNA methylation regulators to influence these key expressions.

In conclusion, for the first time, our study systematically demonstrated the expression and potential functions of m6A RNA methylation regulators in COPD. The expressions of IGF2BP3, FTO, METTL3 and YTHDC2, which have the significant associations with some key genes (such as BCL2A1, GPX2, AKR1B10, ALDH3A1, CABYR, CYP4F3, EGF, UCHL1, CYP1A1, CYP1B1 and MUCL1) enriched in the signalling pathway and biological processes that promote the development progression of COPD, are highly correlated with the occurrence of COPD. In summary, this study provides important evidence for further examination of the role of m6A RNA methylation in COPD. However, future studies are still needed to clarify the detail roles of these RNA methylation regulators and these key genes in COPD, such as how IGF2BP3, FTO, METTL3 and YTHDC2 to influence these genes as well as whether their abnormal interactions lead to the occurrence of COPD.

## CONFLICT OF INTEREST

No conflicts of interest, financial or otherwise, are declared by the authors.

## AUTHOR CONTRIBUTIONS

**Xinwei Huang:** Formal analysis (equal); Resources (equal); Software (equal); Validation (equal); Visualization (equal); Writing‐original draft (equal); Writing‐review & editing (equal). **Dongjin Lv:** Resources (equal); Writing‐original draft (equal). **Xiao Yang:** Data curation (equal). **Min Li:** Data curation (equal). **Hong Zhang:** Writing‐original draft (equal); Writing‐review & editing (equal).

## Supporting information

Fig S1

Fig S2

Fig S3

Fig S4

Table S1‐S2

## Data Availability

The data that support the findings of our study are openly available in the Gene Expression Omnibus (GSE5058, GSE8545, GSE11906 and GSE20257) at https://www.ncbi.nlm.nih.gov/geo/.

## References

[1] Rao W , Wang S , Duleba M , et al. Regenerative metaplastic clones in COPD lung drive inflammation and fibrosis. Cell. 2020;181:848‐864.e18. 32298651 10.1016/j.cell.2020.03.047 PMC7294989

[2] Huang X , Zhu Z , Guo X , Kong X . The roles of microRNAs in the pathogenesis of chronic obstructive pulmonary disease. Int Immunopharmacol. 2019;67:335‐347. 30578969 10.1016/j.intimp.2018.12.013

[3] Tong J , Flavell RA , Li HB . RNA m(6)A modification and its function in diseases. Front Med. 2018;12:481‐489. 30097961 10.1007/s11684-018-0654-8

[4] Wang Q , Zhang H , Chen Q , Wan Z , Gao X . Identification of METTL14 in kidney renal clear cell carcinoma using bioinformatics. Analysis. 2019;2019:5648783. 10.1155/2019/5648783 PMC6954481 31976022

[5] Desrosiers R , Friderici K , Rottman F . Identification of methylated nucleosides in messenger RNA from Novikoff hepatoma cells. Proc Natl Acad Sci USA. 1974;71:3971‐3975. 4372599 10.1073/pnas.71.10.3971 PMC434308

[6] Wu R , Jiang D , Wang Y , Wang X . N (6)‐Methyladenosine (m(6)A) methylation in mRNA with a dynamic and reversible epigenetic modification. Mol Biotechnol. 2016;58:450‐459. 27179969 10.1007/s12033-016-9947-9

[7] Du J , Hou K , Mi S , et al. Malignant evaluation and clinical prognostic values of m6A RNA methylation regulators in glioblastoma. Front Oncol. 2020;10:208. 32211315 10.3389/fonc.2020.00208 PMC7075451

[8] Liu ZX , Li LM , Sun HL , Liu SM . Link between m6A modification and cancers. Front Bioeng Biotech. 2018;6:89. 10.3389/fbioe.2018.00089 PMC6055048 30062093

[9] Li Y , Xiao J , Bai J , et al. Molecular characterization and clinical relevance of m(6)A regulators across 33 cancer types. Mol Cancer. 2019;18:137. 31521193 10.1186/s12943-019-1066-3 PMC6744659

[10] Wu Y , Zhou C , Yuan Q . Role of DNA and RNA N6‐adenine methylation in regulating stem cell fate. Curr Stem Cell Res Ther. 2018;13:31‐38. 28637404 10.2174/1574888X12666170621125457

[11] Yang F , Jin H , Que B , et al. Dynamic m(6)A mRNA methylation reveals the role of METTL3‐m(6)A‐CDCP1 signaling axis in chemical carcinogenesis. Oncogene. 2019;38:4755‐4772. 30796352 10.1038/s41388-019-0755-0 PMC6756049

[12] Liu J , Ren D , Du Z , Wang H , Zhang H , Jin Y . m(6)A demethylase FTO facilitates tumor progression in lung squamous cell carcinoma by regulating MZF1 expression. Biochem Biophys Res Comm. 2018;502:456‐464. 29842885 10.1016/j.bbrc.2018.05.175

[13] Chen M , Wei L , Law CT , et al. RNA N6‐methyladenosine methyltransferase‐like 3 promotes liver cancer progression through YTHDF2‐dependent posttranscriptional silencing of SOCS2. Hepatology. 2018;67:2254‐2270. 29171881 10.1002/hep.29683

[14] Li J , Han Y , Zhang H , et al. The m6A demethylase FTO promotes the growth of lung cancer cells by regulating the m6A level of USP7 mRNA. Biochem Biophys Res Comm. 2019;512:479‐485. 30905413 10.1016/j.bbrc.2019.03.093

[15] Carolan BJ , Heguy A , Harvey BG , Leopold PL , Ferris B , Crystal RG . Up‐regulation of expression of the ubiquitin carboxyl‐terminal hydrolase L1 gene in human airway epithelium of cigarette smokers. Can Res. 2006;66:10729‐10740. 10.1158/0008-5472.CAN-06-2224 17108109

[16] Ammous Z , Hackett NR , Butler MW , et al. Variability in small airway epithelial gene expression among normal smokers. Chest. 2008;133:1344‐1353. 18339782 10.1378/chest.07-2245 PMC3632367

[17] Raman T , O'Connor TP , Hackett NR , et al. Quality control in microarray assessment of gene expression in human airway epithelium. BMC Genom. 2009;10:493. 10.1186/1471-2164-10-493 PMC2774870 19852842

[18] Shaykhiev R , Otaki F , Bonsu P , et al. Cigarette smoking reprograms apical junctional complex molecular architecture in the human airway epithelium in vivo. Cell Mol Life Sci. 2011;68:877‐892. 20820852 10.1007/s00018-010-0500-x PMC3838912

[19] Langfelder P , Horvath S . WGCNA: an R package for weighted correlation network analysis. BMC Bioinformatics. 2008;9:559. 19114008 10.1186/1471-2105-9-559 PMC2631488

[20] Huang X , Li Y , Guo X , Zhu Z , Kong X . Identification of differentially expressed genes and signaling pathways in chronic obstructive pulmonary disease via bioinformatic analysis. FEBS Open Bio. 2019;9:1880‐1899. 10.1002/2211-5463.12719 PMC6823288 31419078

[21] Otasek D , Morris JH , Bouças J , Pico AR , Demchak B . Cytoscape Automation: empowering workflow‐based network analysis. Genome Biol. 2019;20:185. 31477170 10.1186/s13059-019-1758-4 PMC6717989

[22] Zhang JY , Wang Y , Prakash C . Xenobiotic‐metabolizing enzymes in human lung. Curr Drug Metab. 2006;7:939‐948. 17168693 10.2174/138920006779010575

[23] Liu Y , Guo X , Zhao M , et al. Contributions and prognostic values of m(6) A RNA methylation regulators in non‐small‐cell lung cancer. J Cell Physiol. 2020;235(9):6043‐6057. 32052446 10.1002/jcp.29531

[24] Zheng W , Dong X , Zhao Y , et al. Multiple functions and mechanisms underlying the role of METTL3 in human cancers. Front Oncol 2019;9:1403. 31921660 10.3389/fonc.2019.01403 PMC6920212

[25] Dominissini D , Moshitch‐Moshkovitz S , Schwartz S , et al. Topology of the human and mouse m6A RNA methylomes revealed by m6A‐seq. Nature. 2012;485:201‐206. 22575960 10.1038/nature11112

[26] Batista PJ , Molinie B , Wang J , et al. m(6)A RNA modification controls cell fate transition in mammalian embryonic stem cells. Cell Stem Cell. 2014;15:707‐719. 25456834 10.1016/j.stem.2014.09.019 PMC4278749

[27] Wang Y , Li Y , Toth JI , Petroski MD , Zhang Z , Zhao JC . N6‐methyladenosine modification destabilizes developmental regulators in embryonic stem cells. Nat Cell Biol. 2014;16:191‐198. 24394384 10.1038/ncb2902 PMC4640932

[28] Aguilo F , Zhang F , Sancho A , et al. Coordination of m(6)A mRNA methylation and gene transcription by ZFP217 regulates pluripotency and reprogramming. Cell Stem Cell. 2015;17:689‐704. 26526723 10.1016/j.stem.2015.09.005 PMC4671830

[29] Quinlan KG , Verger A , Yaswen P , Crossley M . Amplification of zinc finger gene 217 (ZNF217) and cancer: when good fingers go bad. Biochem Biophys Acta. 2007;1775:333‐340. 17572303 10.1016/j.bbcan.2007.05.001

[30] Chen J , Du B . Novel positioning from obesity to cancer: FTO, an m(6)A RNA demethylase, regulates tumour progression. J Cancer Res Clin Oncol. 2019;145:19‐29. 30465076 10.1007/s00432-018-2796-0 PMC11810187

[31] Adhikari S , Xiao W , Zhao YL , Yang YG . m(6)A: signaling for mRNA splicing. RNA Biol. 2016;13:756‐759. 27351695 10.1080/15476286.2016.1201628 PMC5013988

[32] Kretschmer J , Rao H , Hackert P , Sloan KE , Höbartner C , Bohnsack MT . The m(6)A reader protein YTHDC2 interacts with the small ribosomal subunit and the 5'‐3' exoribonuclease XRN1. RNA. 2018;24:1339‐1350. 29970596 10.1261/rna.064238.117 PMC6140455

[33] Lederer M , Bley N , Schleifer C , Hüttelmaier S . The role of the oncofetal IGF2 mRNA‐binding protein 3 (IGF2BP3) in cancer. Semin Cancer Biol. 2014;29:3‐12. 25068994 10.1016/j.semcancer.2014.07.006

[34] Wu X , Sun X , Chen C , Bai C , Wang X . Dynamic gene expressions of peripheral blood mononuclear cells in patients with acute exacerbation of chronic obstructive pulmonary disease: a preliminary study. Crit Care. 2014;18:508. 25407108 10.1186/s13054-014-0508-y PMC4305227

[35] Mostafaei S , Kazemnejad A , Azimzadeh Jamalkandi S , Amirhashchi S . Identification of novel genes in human airway epithelial cells associated with chronic obstructive pulmonary disease (COPD) using machine‐based learning algorithms. Sci Rep. 2018;8:15775. 30361509 10.1038/s41598-018-33986-8 PMC6202402

[36] Yang D , Yan Y , Hu F , Wang T . CYP1B1, VEGFA, BCL2, and CDKN1A affect the development of chronic obstructive pulmonary disease. Int J Chron Obstruct Pulmon Dis. 2020;15:167‐175. 32158203 10.2147/COPD.S220675 PMC6986178

[37] Steiling K , Lenburg ME , Spira A . Airway gene expression in chronic obstructive pulmonary disease. Proc Am Thorac Soc. 2009;6:697‐700. 20008878 10.1513/pats.200907-076DP PMC2797071

[38] Zhang H , Sun D , Li D , et al. Long non‐coding RNA expression patterns in lung tissues of chronic cigarette smoke induced COPD mouse model. Sci Rep. 2018;8:7609. 29765063 10.1038/s41598-018-25702-3 PMC5954018

[39] Yi G , Liang M , Li M , et al. A large lung gene expression study identifying IL1B as a novel player in airway inflammation in COPD airway epithelial cells. Inflamm Res. 2018;67:539‐551. 29616282 10.1007/s00011-018-1145-8

[40] de Boer WI , Hau CM , van Schadewijk A , Stolk J , van Krieken JH , Hiemstra PS . Expression of epidermal growth factors and their receptors in the bronchial epithelium of subjects with chronic obstructive pulmonary disease. Am J Clin Pathol. 2006;125:184‐192. 16393673 10.1309/W1AX-KGT7-UA37-X257

[41] Awji EG , Chand H , Bruse S , et al. Wood smoke enhances cigarette smoke‐induced inflammation by inducing the aryl hydrocarbon receptor repressor in airway epithelial cells. Am J Respir Cell Mol Biol. 2015;52:377‐386. 25137396 10.1165/rcmb.2014-0142OC PMC4370262

[42] Jang JH , Bruse S , Liu Y , et al. Aldehyde dehydrogenase 3A1 protects airway epithelial cells from cigarette smoke‐induced DNA damage and cytotoxicity. Free Radic Biol Med. 2014;68:80‐86. 24316006 10.1016/j.freeradbiomed.2013.11.028 PMC3941192

[43] Soemarwoto RA , Jamsari Y , Putra AC , Mustofa S . Decreased plasma epidermal growth factor (EGF) levels in patients with severe chronic obstructive pulmonary disease. Pneumologia. 2019;68(1):21.

[44] Wortham BW , Eppert BL , Flury JL , Garcia SM , Donica WR , Osterburg A . Cutting edge: CLEC5A mediates macrophage function and chronic obstructive pulmonary disease pathologies. J Immunol. 1950;2016(196):3227‐3231. 10.4049/jimmunol.1500978 PMC4821706 26927798

[45] Woodruff PG , Ellwanger A , Solon M , Cambier CJ , Pinkerton KE , Koth LL . Alveolar macrophage recruitment and activation by chronic second hand smoke exposure in mice. COPD. 2009;6:86‐94. 19378221 10.1080/15412550902751738 PMC2873864

[46] Shan M , Yuan X , Song LZ , et al. Cigarette smoke induction of osteopontin (SPP1) mediates T(H)17 inflammation in human and experimental emphysema. Sci Transl Med. 2012;4(117):117ra9. 10.1126/scitranslmed.3003041 PMC3956594 22261033

[47] Korytina GF , Akhmadishina LZ , Viktorova TV . The CYP1B1 and CYP2F1 genes polymorphisms frequency in three ethnic groups of Bashkortostan and chronic obstructive pulmonary disease patients. Mol Biol. 2010;44:33‐41. 20198857

[48] Hukkanen J , Pelkonen O , Hakkola J , Raunio H . Expression and regulation of xenobiotic‐metabolizing cytochrome P450 (CYP) enzymes in human lung. Crit Rev Toxicol. 2002;32:391‐411. 12389869 10.1080/20024091064273

