# Supplementary figures and images for: m6A RNA methylation regulators could contribute to the occurrence of chronic obstructive pulmonary disease
Source: J Cell Mol Med. 2020 Sep 22;24(21):12706–15. doi: 10.1111/jcmm.15848 (PMC7686997; doi:10.1111/jcmm.15848)

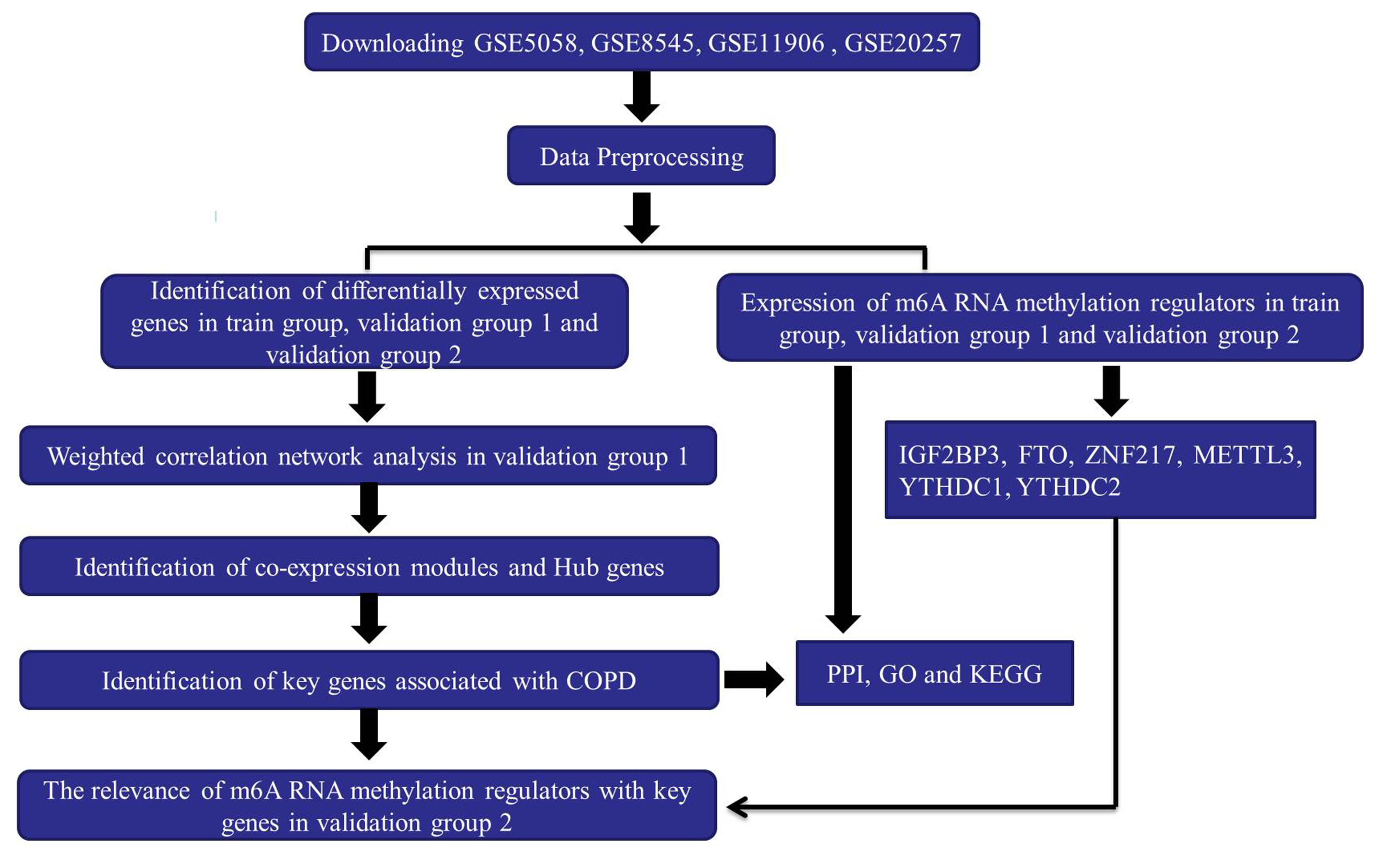

Supplement: Supplementary file 1 — Fig S1 [file JCMM-24-12706-s001.jpg]

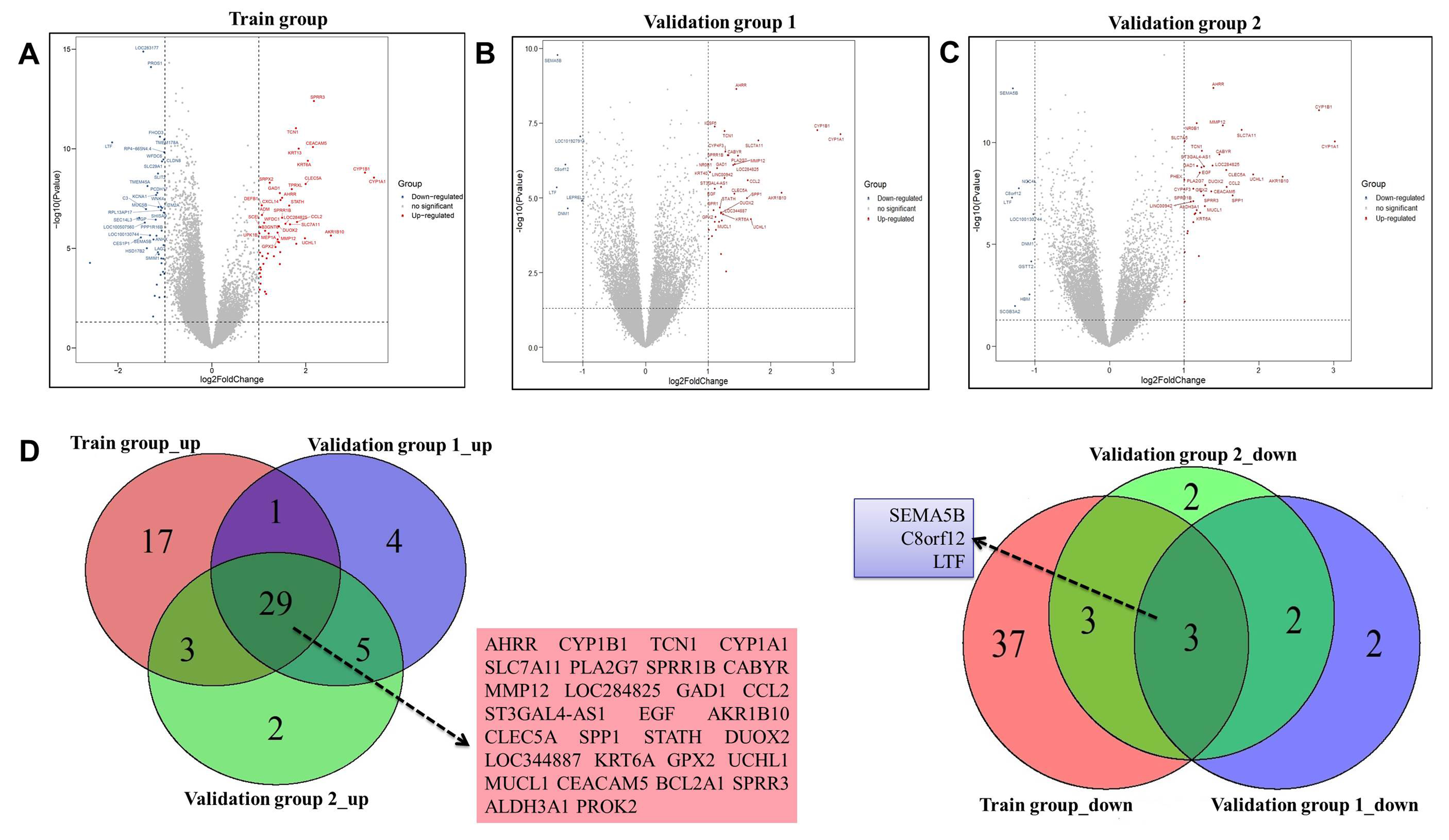

Supplement: Supplementary file 2 — Fig S2 [file JCMM-24-12706-s002.jpg]

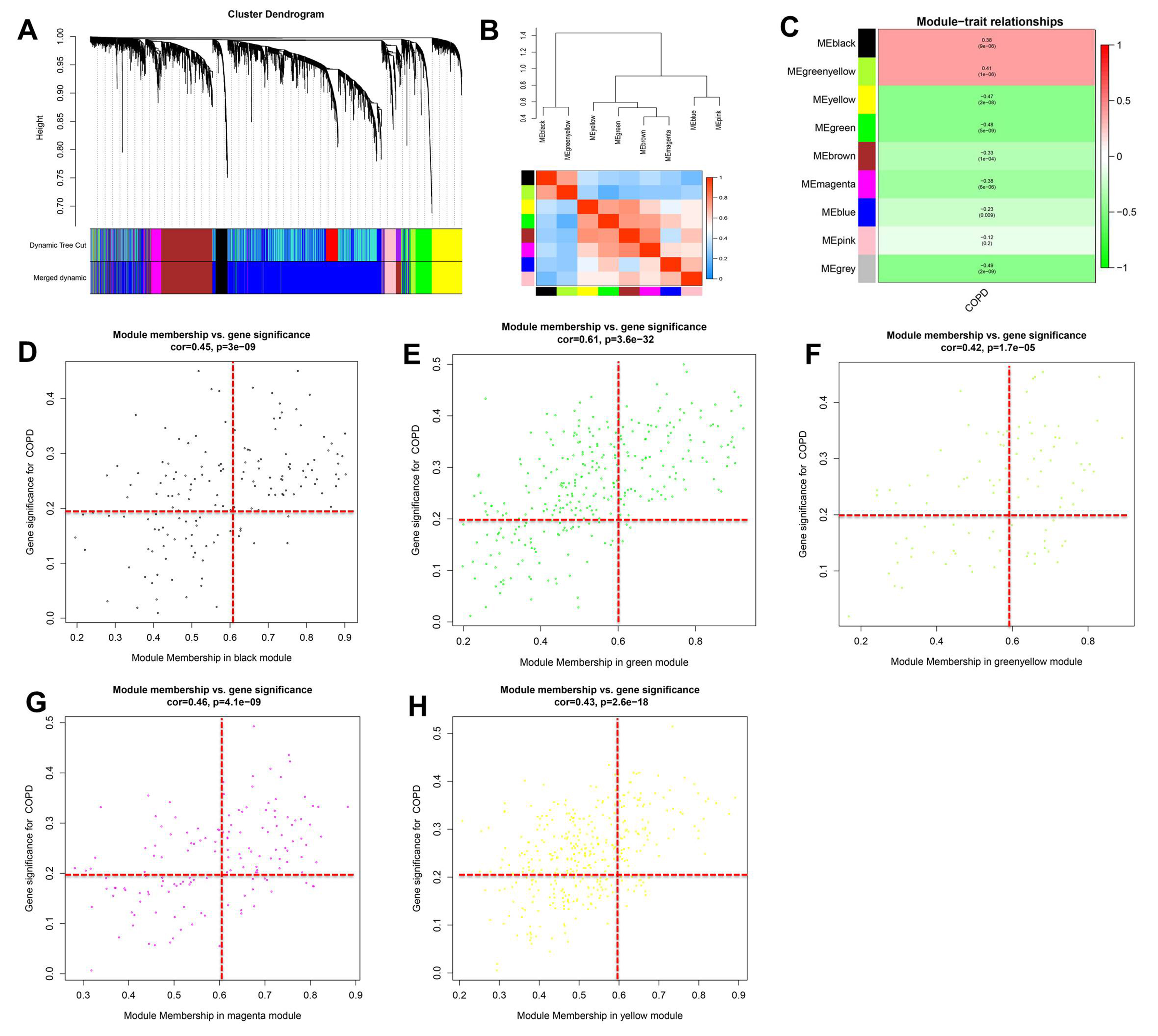

Supplement: Supplementary file 3 — Fig S3 [file JCMM-24-12706-s003.jpg]

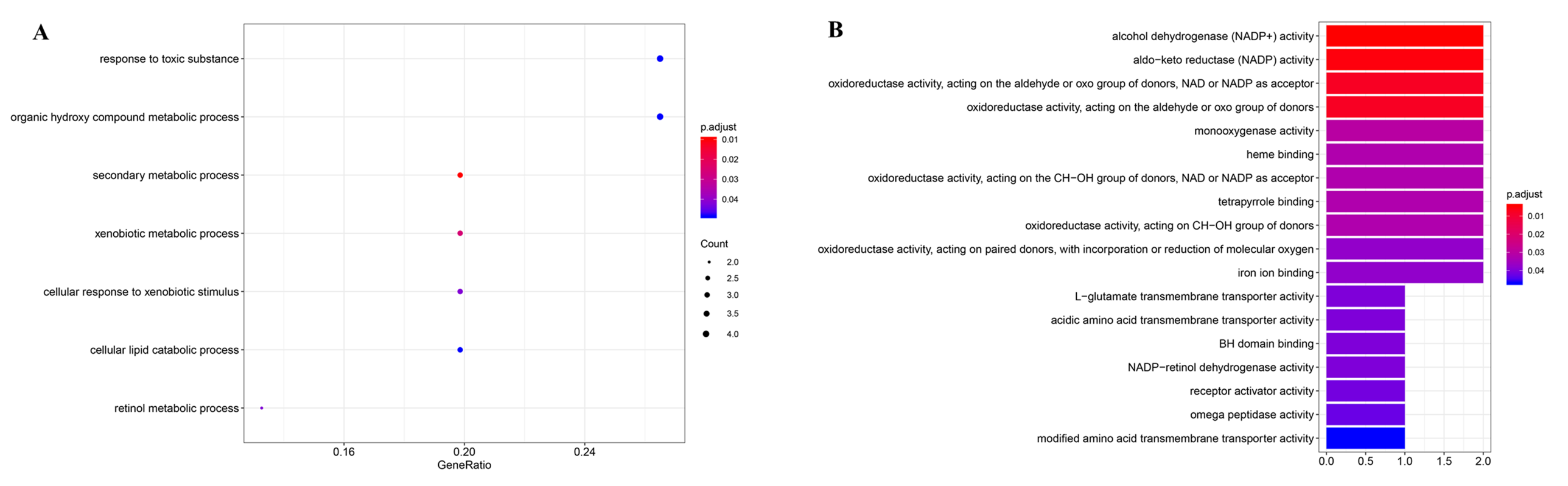

Supplement: Supplementary file 4 — Fig S4 [file JCMM-24-12706-s004.jpg]
