# Supplementary material for: m6A RNA methylation regulators could contribute to the occurrence of chronic obstructive pulmonary disease
Source: J Cell Mol Med. 2020 Sep 22;24(21):12706–15. doi: 10.1111/jcmm.15848 (PMC7686997; doi:10.1111/jcmm.15848)
Supplement: Supplementary file 5 — Table S1‐S2 [file JCMM-24-12706-s005.docx]

**Supplementary Table 1** GO function analyses for proteins interacting with IGF2BP3, FTO, ZNF217, METTL3, YTHDC1, and YTHDC2.

| Category | Term | Description | Count | P Value |
| --- | --- | --- | --- | --- |
| **IGF2BP3** |  |  |  |  |
| Biological process | GO:0010467 | gene expression | 8/24 | 1.11E-13 |
| Biological process | GO:0080009 | mRNA methylation | 4/24 | 3.36E-08 |
| Biological process | GO:0006397 | mRNA processing | 6/24 | 2.31E-06 |
| Biological process | GO:0000398 | mRNA splicing, via spliceosome | 5/24 | 0.000149 |
| Biological process | GO:1903679 | positive regulation of cap-independent translational initiation | 2/24 | 0.003747 |
| Biological process | GO:0061157 | mRNA destabilization | 2/24 | 0.007481 |
| Biological process | GO:0031053 | primary miRNA processing | 2/24 | 0.011202 |
| Molecular function | GO:0003729 | mRNA binding | 5/24 | 2.27E-05 |
| Molecular function | GO:0016422 | mRNA (2'-O-methyladenosine-N6-)-methyltransferase activity | 2/24 | 0.002723 |
| Molecular function | GO:1904047 | S-adenosyl-L-methionine binding | 2/24 | 0.006795 |
| Molecular function | GO:0008168 | methyltransferase activity | 3/24 | 0.007193 |
| **METTL3** |  |  |  |  |
| Biological process | GO:0010467 | gene expression | 8/24 | 1.11E-13 |
| Biological process | GO:0042035 | regulation of cytokine biosynthetic process | 3/24 | 3.12E-05 |
| Biological process | GO:0051028 | mRNA transport | 3/24 | 0.001557 |
| Biological process | GO:0017148 | negative regulation of translation | 3/24 | 0.002361 |
| Molecular function | GO:0044822 | poly(A) RNA binding | 16/24 | 6.46E-13 |
| Molecular function | GO:0003723 | RNA binding | 10/24 | 2.01E-08 |
| Molecular function | GO:0000166 | nucleotide binding | 8/24 | 2.75E-07 |
| Molecular function | GO:0003730 | mRNA 3'-UTR binding | 5/24 | 5.33E-07 |
| Molecular function | GO:0045182 | translation regulator activity | 3/24 | 2.65E-05 |
| Molecular function | GO:0003676 | nucleic acid binding | 9/24 | 2.91E-05 |
| Molecular function | GO:0048027 | mRNA 5'-UTR binding | 3/24 | 6.36E-05 |
| **FTO** |  |  |  |  |
| Biological process | GO:0035553 | oxidative single-stranded RNA demethylation | 2/24 | 0.003747 |
| Molecular function | GO:0035515 | oxidative RNA demethylase activity | 2/24 | 0.002723 |
| **ZNF217** |  |  |  |  |
| Molecular function | GO:0003676 | nucleic acid binding | 9/24 | 2.91E-05 |
| Molecular function | GO:0005515 | protein binding | 18/24 | 0.027506 |
| **YTHDC1** | GO:1990247 | N6-methyladenosine-containing RNA binding | 7/24 | 2.20E-17 |
| Molecular function | GO:0044822 | poly(A) RNA binding | 16/24 | 6.46E-13 |
| Molecular function | GO:0003723 | RNA binding | 10/24 | 2.01E-08 |
| Molecular function | GO:0005515 | protein binding | 18/24 | 0.027506 |
| **YTHDC2** |  |  |  |  |
| Molecular function | GO:1990247 | N6-methyladenosine-containing RNA binding | 7/24 | 2.20E-17 |
| Molecular function | GO:0044822 | poly(A) RNA binding | 16/24 | 6.46E-13 |
| Molecular function | GO:0003676 | nucleic acid binding | 9/24 | 2.91E-05 |
| Molecular function | GO:0005515 | protein binding | 18/24 | 0.027506 |

Note: GO, Gene Ontology.

**Supplementary Table 2** The mRNA expression levels of 27 key COPD genes in a variety of samples of chronic obstructive pulmonary disease.

| Genes | Function (<https://www.genecards.org/>) | Group | Sample type | The mRNA expression level | Ref |
| --- | --- | --- | --- | --- | --- |
| BCL2A1 | Maintains endothelial survival and inhibits apoptosis | Acute exacerbation COPD vs. stable COPD | Peripheral blood mononuclear cells | Up | [^34^](#_ENREF_34) |
| AKR1B10 | Detoxifies dietary and lipid-derived unsaturated carbonyls | Smokers with COPD vs. healthy smokers | Large airway epithelium | Up | [^37^](#_ENREF_37) |
| CABYR | Regulates protein tyrosine phosphorylation and intracellular calcium | COPD vs. control | small airway epithelium | Up | [^35^](#_ENREF_35) |
| GPX2 | Plays a major role in protecting mammals from the toxicity of ingested organic hydroperoxides | Smokers with COPD vs. healthy smokers | Large airway epithelium | Up | [^37^](#_ENREF_37) |
| SLC7A11 | Promotes cystine uptake and glutathione biosynthesis, resulting in protection from oxidative stress and ferroptotic cell death | COPD vs. control | small airway epithelium | Up | [^35^](#_ENREF_35) |
| MUCL1 | Serve as an attractive tumor-associated antigen | COPD vs. control | small airway epithelium | Up | [^35^](#_ENREF_35) |
| CYP1B1 | Catalyzes the oxidative metabolism of exogenous and endogenous substrates | Smokers with COPD vs. healthy smokers | Large airway epithelium; small airway epithelium; quadriceps; blood | Up | [^35^](#_ENREF_35)^,^ [^37^](#_ENREF_37) [^36^](#_ENREF_36)^,^ [^48^](#_ENREF_48)^,^ [^49^](#_ENREF_49) |
| UCHL1 | Regulates the degradation of unwanted, damaged, or misfolded proteins | COPD vs. control | PBMCs of human; the lung of mouse; small airway epithelium | Up | [^15^](#_ENREF_15)^,^ [^35^](#_ENREF_35)^,^ [^38^](#_ENREF_38) |
| ST3GAL4-AS1 | Long non-coding RNA gene associated with colorectal cancer and Al-raqad syndrome | COPD vs. control | small airway epithelium | Up | [^35^](#_ENREF_35) |
| ALDH3A1 | Catalyzes the oxidation of reactive aldehydes | COPD vs. control | The lung | Up | [^39^](#_ENREF_39)^,^ [^42^](#_ENREF_42) |
| LOC344887 | Undefined | COPD vs. control | Small airway epithelium | Up | [^35^](#_ENREF_35) |
| AHRR | Mediates environmental toxicant-induced inflammation | COPD vs. control | Small airway epithelium | Up | [^35^](#_ENREF_35)^,^ [^41^](#_ENREF_41) |
| EGF | Modulates airway hyperproliferation and increase mucus hyperproduction and mucous cell differentiation | Ex-smokers with COPD vs. ex-smokers | Epithelium | Up | [^40^](#_ENREF_40)^,^ [^43^](#_ENREF_43) |
| SEMA5B | Acts as positive axonal guidance cues. | - | - | - | - |
| LOC284825 | Undefined | - | - | - | - |
| CLEC5A | CLEC5A is required for the development of inflammation, proinflammatory cytokine expression and airspace enlargement | CS-exposure mice vs. FA -exposed mice | Lung macrophage | Up | [^44^](#_ENREF_44) |
| CCL2 | Induces a strong chemotactic response and mobilization of intracellular calcium ions | COPD vs. control | Lung small airway samples | Up | [^39^](#_ENREF_39) |
| MMP12 | May be involved in tissue injury and remodeling | COPD vs. control | Lung small airway samples | Up | [^39^](#_ENREF_39) |
| PROK2 | Functions as an output molecule from the suprachiasmatic nucleus (SCN) that transmits behavioral circadian rhythm | - | - | - | - |
| PLA2G7 | Plays an important role in atherosclerosis by oxidizing lipids into reactive intermediates | Smoke-exposed mice vs. air-exposed mice | Alveolar macrophage | Up | [^45^](#_ENREF_45) |
| GAD1 | - | Smokers with COPD vs. healthy smokers | Small airway epithelium | Up | [^35^](#_ENREF_35) |
| CYP1A1 | A cytochrome P450 monooxygenase involved in the metabolism of various endogenous substrates | Smokers with COPD vs. healthy smokers | Small airway epithelium | Up | [^35^](#_ENREF_35) |
| SPP1 | Acts as a cytokine involved in enhancing production of interferon-gamma | Smoke-exposed mice vs. air-exposed mice | Antigen-presenting cells | Up | [^47^](#_ENREF_47) |
| LTF | Stimulates the TLR4 signaling pathway leading to NF-kappa-B activation and subsequent pro-inflammatory cytokine production | - | - | - | - |
| STATH | Modulates hydroxyapatite crystal formation on the tooth surface | - | - | - | - |
| TCN1 | Binds vitamin B12 with femtomolar affinity and protects it from the acidic environment of the stomach | - | - | - | - |
| CEACAM5 | Mediates homophilic and heterophilic cell adhesion | - | - | - | - |

Note: COPD, chronic obstructive pulmonary disease.
